# Epigenetic Regulation of Endothelial Cell Lineages During Zebrafish Development—New Insights From Technical Advances

**DOI:** 10.3389/fcell.2022.891538

**Published:** 2022-05-09

**Authors:** Virginia Panara, Rui Monteiro, Katarzyna Koltowska

**Affiliations:** ^1^ Immunology Genetics and Pathology, Uppsala University, Uppsala, Sweden; ^2^ Institute of Cancer and Genomic Sciences, College of Medical and Dental Sciences, University of Birmingham, Birmingham, United Kingdom; ^3^ Birmingham Centre of Genome Biology, University of Birmingham, Birmingham, United Kingdom

**Keywords:** blood endothelial cells, lymphatic endothelial cells, epigenetics, ATAC-seq, ChIP-seq, enhancers, conserved non-coding elements, chromatin structure

## Abstract

Epigenetic regulation is integral in orchestrating the spatiotemporal regulation of gene expression which underlies tissue development. The emergence of new tools to assess genome-wide epigenetic modifications has enabled significant advances in the field of vascular biology in zebrafish. Zebrafish represents a powerful model to investigate the activity of *cis*-regulatory elements *in vivo* by combining technologies such as ATAC-seq, ChIP-seq and CUT&Tag with the generation of transgenic lines and live imaging to validate the activity of these regulatory elements. Recently, this approach led to the identification and characterization of key enhancers of important vascular genes, such as *gata2a, notch1b* and *dll4*. In this review we will discuss how the latest technologies in epigenetics are being used in the zebrafish to determine chromatin states and assess the function of the *cis*-regulatory sequences that shape the zebrafish vascular network.

## Introduction

In a developing embryo, a sequence of events guides the transformation of undifferentiated cells to become specialized functional tissues and organs. This process requires the synchronization of a multitude of factors in time and space to orchestrate cellular processes such as proliferation, differentiation, migration and survival. One of the key factors in the acquisition of cell identity is differential gene expression. All cells in an organism largely share the same DNA sequence, yet they activate and repress specific gene expression to acquire lineage-specific morphologies and functionalities. This is achieved largely due to epigenetic changes, including DNA and chromatin modification and non-coding RNA regulation, allowing for tissue specific expression.

Endothelial cells (ECs) constitute the lining of the blood and lymphatic vascular networks that in vertebrates reach all the tissues and organs across the body. These vascular networks are essential to support life by delivering oxygen and nutrients, removing waste, maintaining fluid homeostasis and facilitating immune functions. During embryonic development, the forming blood and lymphatic vascular networks undergo a cellular and molecular transformation to generate functionally and morphologically distinct entities that support their specific functions. This requires the synchronization of morphological changes with the onset of lineage-specific gene expression. This is achieved by a combination of gene sets that provide a specific molecular code for each lineage (reviewed in Yamashita, 2007; Kume, 2010; Wolf et al., 2019). Epigenetic regulation, and in particular chromatin organization, dictates the accessibility for transcription factors to bind the DNA sequences in the non-coding regions and mediate lineage specific gene expression (Lickert et al., 2004; Wu et al., 2007; Alvarez-Saavedra et al., 2014).

Thanks to the technological advances in recent years, we are gaining deeper insights into the epigenetic regulation of vascular identity. This review will focus on the application of these new techniques to define the spatiotemporal regulation of endothelial lineages in zebrafish. We will discuss three different and complementary approaches for the characterization of epigenetic regulation of endothelial gene expression in zebrafish: chromatin state, mapping of histone modifications and conservation of non-coding elements. As each of these approaches provide different information about gene expression regulation, their combination can be used to obtain a precise and accurate prediction of the presence of *cis*-regulatory elements, as well as their activation state in specific times and cell-lineages and their direct upstream regulation. Since non-coding RNAs in zebrafish and endothelial cells have been recently extensively reviewed (Weirick et al., 2018; Jaé et al., 2019; Ranjan et al., 2021) we will focus on other aspects of epigenetic regulation, such as *cis*-regulatory elements, histone modifications and 3D genomic architecture. We will also briefly review the main strategies used to generate enhancer reporter lines in zebrafish, an organism particularly suitable for rapid and accurate testing of enhancer function and activity *in vivo*, due to the ease of transgenesis and live imaging.

## Transcriptional Regulation of Endothelial Cell Lineages

**FIGURE 1:**
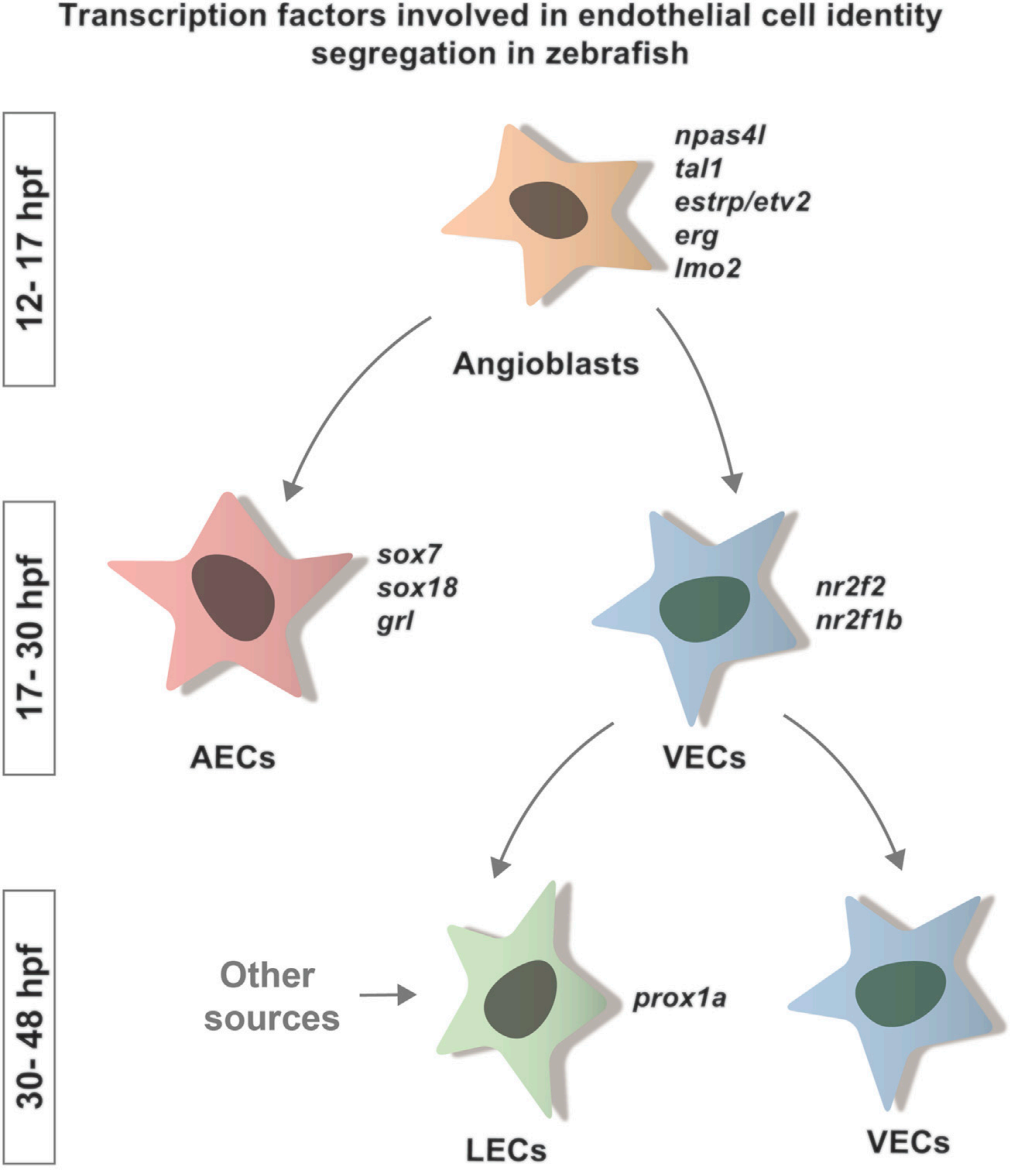
Transcription factors involved in endothelial cell identity segregation in zebrafish. Schematic representation of the specification of the main ECs lineages and the TFs involved in the acquisition of their identity. Many factors are known to be involved in this process in zebrafish. Here, we indicate the TFs known to play a role in the segregation of arterial (*sox7*, *sox18*, *grl*) and venous (*nr2f2*, *nr2f1b*) identity. So far, the only TF linked to LEC identity segregation in zebrafish is *prox1a*. These factors bind to region of open chromatin to promote the acquisition of a specific cellular fate.

In zebrafish, ECs originate from the lateral plate mesoderm (LPM) which, by a sequence of differentiation events, gives rise to specialized endothelial cell types, the arterial ECs (AECs), venous ECs (VECs) and lymphatic ECs (LECs) (Hogan and Schulte-Merker, 2017) (Figure 1). During these complex developmental processes, several transcription factors (TFs) play a key role in orchestrating the segregation and identity acquisition of the different lineages.

During the early stages of EC specification, the transcription factor Npas4l regulates the acquisition of angioblast identity (Stainier et al., 1995; Reischauer et al., 2016) through the expression of other TFs, such as *erg* (Ellett et al., 2009), *etsrp/etv2* (Sumanas and Lin, 2005; Veldman and Lin, 2012; Reischauer et al., 2016; Marass et al., 2019), *tal1* (Gering et al., 2003; Patterson et al., 2007; Veldman and Lin, 2012; Reischauer et al., 2016; Marass et al., 2019), *lmo2* (Gering et al., 2003; Patterson et al., 2007; Marass et al., 2019) and *fli1a* (Thompson et al., 1998; Reischauer et al., 2016) (Figure 1).

As development proceeds, the fate of the arterial and venous lineage begins to segregate. Although upstream signaling, such as Notch and Efnb2/ephB4, is an important modulator of these processes, we focus on the TFs involved in the segregation, as their activity is linked to chromatin accessibility. AECs identity is induced by transcription factors including Sox7, Sox18 (Herpers et al., 2008; Pendeville et al., 2008) and *grl* (Zhong, 2000). Transcription factors such as Nr2f2/COUPTFII (Aranguren et al., 2011) and *nr2f1b* (Li et al., 2015) are required to define the venous population (Figure 1). Foxc1a and Foxc1b are also necessary for the correct arteriovenous differentiation (Skarie and Link, 2009). A specific role for SMAD1/5 in the acquisition of venous identity has also been proposed by comparative work on mouse and zebrafish (Neal et al., 2019).

The last endothelial population to differentiate are the LECs, which originates from the cardinal veins. Its differentiation is marked by the expression of the transcription factor Prox1a (Koltowska et al., 2015a) (Figure 1). LEC progenitors form a functionally distinct vasculature, the lymphatic vascular network (Küchler et al., 2006; Yaniv et al., 2006; Hogan et al., 2009a).

As we have seen, TFs play a key role in orchestrating the differentiation of endothelial cell populations. As many of them are expressed in wider cell populations than the one they regulate, the endothelial specification processes must therefore be accompanied by an underlying re-organization of the genomic DNA. Such re-organization changes the epigenetic landscape making different regulatory DNA sequences accessible to lineage-specific TFs. However, in which specific ways the genome is reorganized in endothelial cell lineages to coordinate the activation of specific gene sets is still a largely unexplored and fascinating question.

## DNA Organization and Enhancers

**FIGURE 2:**
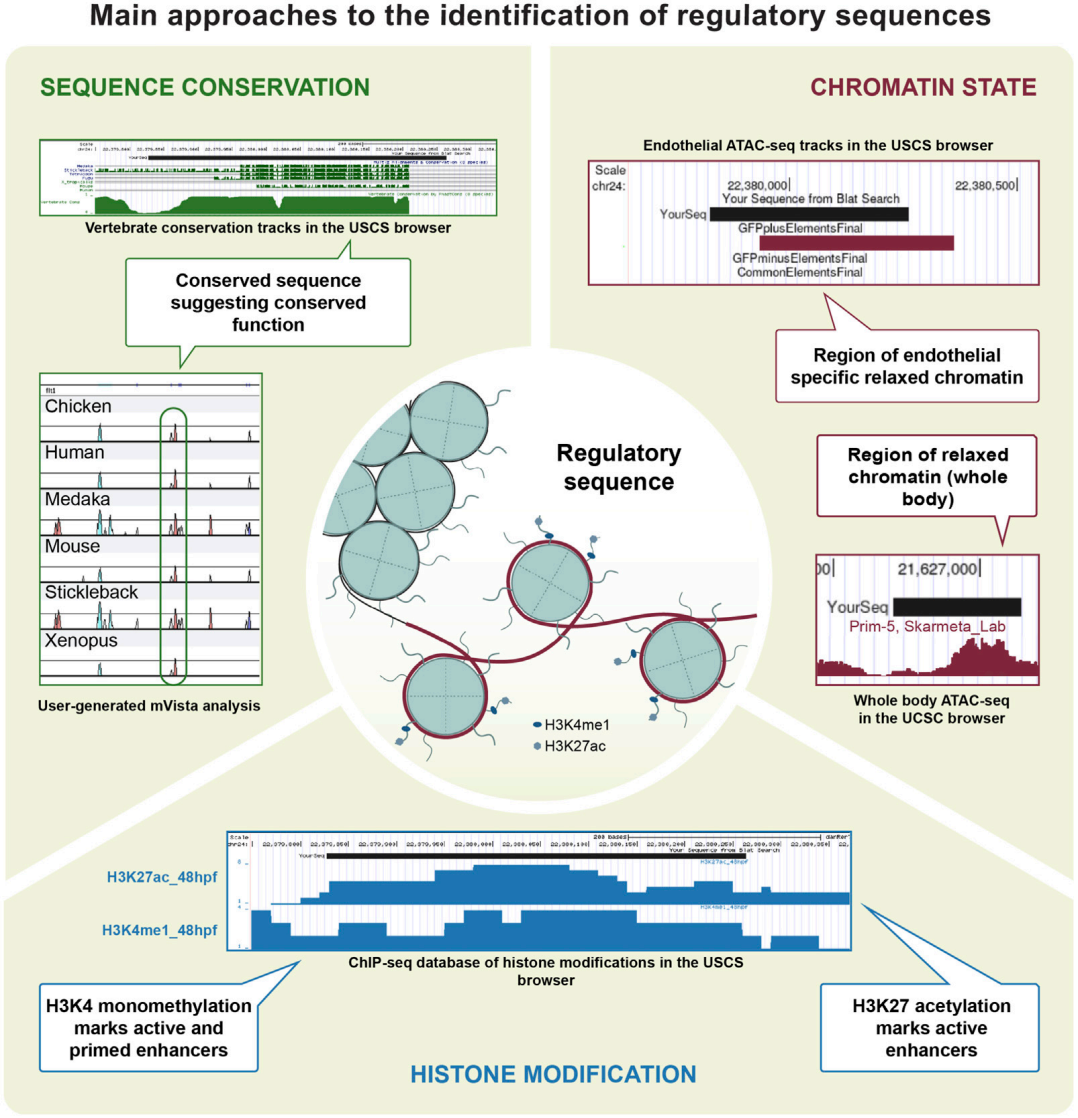
Main approaches to the identification of regulatory sequences. Technical approaches for the identification and characterisation of CREs. Zebrafish EC regulatory sequences have been identified based on conservation, as is the case for genes such as *flt1*, *etsrp*, *gata2a* and *notch1*. Chromatin accessibility was also successfully investigated in the EC population, contributing to the identifications of CREs in *gata2a* and *mafbb*. Data on histone modification was generated using ChIP-seq and CUT&RUN on EC in other organisms, and is available for zebrafish in a variety of tissues.

The DNA that is present in every cell nucleus is not a free molecule, but is wrapped around histones to form a structure called chromatin. In addition to providing protection from damage to the DNA, chromatin helps determine whether DNA is easily made accessible (or inaccessible) for gene regulation. Chromatin organization is a highly dynamic process: upon acetylation or methylation of the histones, the DNA can become more relaxed (euchromatin) or be pulled tightly into an interlocked, organized bundle (heterochromatin) (Figure 2). Likewise, DNA methylation can negatively regulate gene activity by preventing the binding of TFs and recruiting transcriptional repressors (Moore et al., 2013). The chromatin state is central to gene activity, as the dogma postulates that open chromatin is associated with active gene expression, allowing the RNA-polymerase complex and the transcription factors to interact with their binding sites on the exposed regions of DNA, called *cis*-regulatory sequences (CREs).

Enhancers are a subclass of CREs and can be located both upstream and downstream of the transcription start sites. As such, enhancers do not contain the sequences necessary for RNA-polymerase recruitment, but can regulate expression by being brought in close proximity to the translational machine by a DNA loop (Su et al., 1990). In addition, gene expression can also be regulated by long-acting enhancers through chromatin looping that brings together distant regions of DNA. While the short-range enhancers act in *cis*, where the regulatory element and its target are located on the same chromosome, the long-range enhancers can act both in *cis* and in *trans* (Bashkirova and Lomvardas, 2019; Tomikawa et al., 2020). This requires 3D folding of the chromatin to bring the regulatory element located on a different chromosome close to the target locus. Often more than one enhancer regulates expression of a gene in different tissues across different developmental stages (Long et al., 2016). From an evolutionary point of view, this has allowed the developmental regulation in a tissue to undergo changes without affecting the expression of the same gene in other parts of the embryo. Thus, enhancers are particularly interesting elements to study in the developmental biology field, as they provide the key to spatio-temporal regulation of gene expression.

## Defining the Chromatin State of Endothelial Cells

As transcriptionally active elements are associated with euchromatin, and inactive ones with heterochromatin, mapping and comparing chromatin states in specific cell populations allows us to identify regions and elements underpinning the lineage specific regulation of gene expression, such as active promoters and enhancers.

Historically, DNAse hypersensitivity has been the method used to assess chromatin state. This technique takes advantage of the ability of the DNAse I enzyme to cleave exposed regions of chromatin to identify potential CREs. Combining the DNAse genomic DNA cuts with tagging and sequencing of the short fragments using a next-generation sequencing platform allows to identify genomic regions of open chromatin. DNAse hypersensitivity databases have been generated for human and other mammals (Boyle et al., 2008; Vierstra et al., 2014). Although it has been used on human Umbilical Vein Endothelial cells (HUVECs), the association of open chromatin regions with specific endothelial genes has not been described in details (Song and Crawford, 2010; Winter et al., 2013). The lack of DNAse hypersensitivity databases for zebrafish or other teleost fishes and the high cell numbers required for DNAse-seq make it a challenging approach for studying tissue specific regulation in these organisms and contributed to its low popularity among zebrafish vascular biologists.

An alternative approach to identify the open chromatin regions is the Assay for Transposase-Accessible Chromatin with high-throughput sequencing (ATAC-seq) (Buenrostro et al., 2013). This technique relies on a hyperactive Tn5 transposase and the loading of sequencing adapters in the areas lacking histones. Thanks to the low cell number input required and the technical ease, ATAC-seq has become a favourite method in the field, and has been readily used in vascular biology (Table 1). ATAC-seq provides a variety of information on chromatin organization. It allows the identification of putatively active genes, marked by significantly different chromatin accessibility around the transcription starting sites (TSS) (Buenrostro et al., 2013). In addition, the distal peaks of open chromatin in the non-coding regions of a locus provide insights into the presence of potential *cis*-regulatory elements (Cusanovich et al., 2018; Galang et al., 2020). This technology can be used to characterize the changes in chromatin architecture between normal and pathological conditions (Corces et al., 2016, 2018). In the last years, new analysis methods allowing the characterization of regulatory networks from ATAC-seq data, based on either motifs (Tripodi et al., 2018; Zuo et al., 2019) or TFs footprints (Quach and Furey, 2016; Baek et al., 2017; Pranzatelli et al., 2018; Li et al., 2019), have also been developed.

**TABLE 1:** Available datasets for ECs in mammals. Summary of the available ATAC-seq, histone modification ChIP-seq and CAGE-seq studies in murine and human endothelial cells.

Technique	Organism/Cell line	Endothelial	Stage	Reference	Enhancers identified
ATAC-seq studies on endothelial polulations in mouse and human					
ATAC-seq	Mouse	Aortic Ecs	Young adult	Engelbrecht et al. (2020)	Genome-wide *in silico* predictions
ATAC-seq	Human primary cells	HUVECs	—	Findley et al. (2019)	—
ATAC-seq	Mouse	Liver sinusoidal ECs	Adult	Furuta et al. (2021)	—
ATAC-seq	Cell line	HUVECs	—	Han et al., (2021)	—
ATAC-seq	Cell line	HUVECs	—	He et al. (2019)	—
ATAC-seq	Human primary cells	Aortic ECs	—	Hogan et al. (2017)	Genome-wide *in silico* predictions
ATAC-seq	Cell line, differentiated *in vitro*	Hemogenic endotheliium	—	Jung et al. (2021)	—
ATAC-seq	Cell line	Aortic Ecs	—	Krause et al. (2018)	Endothelial enhancer in the *Plpp3* intronic region
ATAC-seq	Cell line	HUVECs	—	Leisegang et al. (2017)	—
ATAC-seq	Human biopsy	Vascular tissue	—	Li et al. (2020)	—
ATAC-seq	Mouse	Brain, liver, lung and kidney ECs	P7	Sabbagh et al. (2018)	Genome-wide *in silico* predictions
ATAC-seq	Mouse primary cells	Brain Ecs	Young	Sabbagh and Nathans, (2020)	—
ATAC-seq	Human primary cells	Aortic ECs	—	Stolze et al, (2020)	*Kif26b*, *Fgd6* and *Vegfc* enhancres characterized *in vitro*
ATAC-seq	Human primary cells	Dermal Ecs	—	Tsou et al. (2016)	—
ATAC-seq	Human primary cells	Dermal Ecs	—	Tsou et al. (2021)	—
ATAC-seq	Mouse	Liver sinusoidal ECs	Adult	Winkler et al. (2021)	—
ATAC-seq	Mouse	Retinal ECs	P6	Yanagida et al. (2020)	—
ATAC-seq	Mouse	Endocardium	Adult	Yang et al. (2020)	—
scATAC-seq	Mouse	Carotid AECs	Adult	Ando et al. (2016)	—
scATAC-seq	Mouse	Ecs from 13 different organs	Adult	Cusanovich et al. (2018)	—
scATAC-seq	Human biopsy	ECs component of carotid arteriosclerotic plaques	—	Depuydt et al. (2020)	—
scATAC-seq	Human fetal tissue	ECs from 15 organs	89–125 days	Domcke et al. (2020)	—
scATAC-seq	Mouse	ECs	E8.25	Pijuan-sala et al. (2020)	Flt1 +67 kb; Maml3 +360 kb
Histone modification studies in mouse endothelial cells					
ChIP-seq on H3K4me3, H3K9ac,H3K27ac, and H3K27me3	Differentiated mouse SCs	Haemogenic endothelium	—	Goode et al. (2016)	—
ChIP-seq on H3K4me1	Mouse	ECs	E12.5	Harada et al. (2021)	2 *Sgk1* enhancers
ChIP-seq on H3K27me3 and H3K4me3	Differentiated mouse	ECs	—	Kanki et al. (2017)	Genome-wide *in silico* prediction
ChIP-seq on H3K27ac/H3K4me3	SCs Mouse	Liver sinusoidal ECs	Adult	Winkler et al. (2021)	—
CAGE-seq studies on endothelial cells					
CAGE-seq	Human and Mouse	Various	—	The FANTOM Consortium and the RIKEN PMI and CLST (DGT), 2014	—
CAGE-seq	Human primary cells	Dermal LECs and BECs	—	Dieterich et al. (2015)	—
CAGE-seq	Human primary cells (reanalysis of the data from Dietrich et al., 2015) Dermal LECs and BECs	Dermal LECs and nBECs	—	Dieterich et al. (2017)	—
CAGE-seq	Human (coltured cells)	Dermal LECs and BECs	Neonatal	Ducoli et al. (2021)	—

In zebrafish, ATAC-seq studies defining the changes of chromatin organization in endothelial cells through development are rapidly emerging. Quillien et al. used Fluorescent Activated Nuclei Sorting (FANS) followed by ATAC-seq to identify a number of endothelial-specific open chromatin regions in zebrafish at 24 hpf (Table 2). They did so by taking advantage of the *Tg(fli1a:egfp)* ^*y1*^ line and compared chromatin states between GFP-labelled endothelial cells and GFP-negative, non-endothelial ones. They identified about 5,000 enhancer elements that were enriched specifically in endothelial cells. They have validated their predictions by confirming the functionality of 9 out of 12 tested elements, which were able to drive GFP expression in the endothelium of transgenic zebrafish embryos (Quillien et al., 2017).

**TABLE 2:** Available datasets for EC enhancer identification in zebrafish. Summary of the available ATAC-seq and histone modification ChIP-seq studies which can be used to investigate the presence of endothelial enhancers.

**Technique**	**Tissue**	**Stage**	**Genotypes**	**Translegic line**	**Reference**
ATAC-seq studies					
ATAC-seq	Endothelium	24 hpf	WT	Tg(fli1a:egfp)^y1^	Quillien et al. (2017)
ATAC-seq	Endothelium	26 hpf	WT	Tg(kdrl:GFP)^s843^	Dobrzycki et al. (2020)
ATAC-seq	Endothelium	29 hpf	WT	TgBAC(runx1P2:Citrine); Tg(kdrl:mCherry)	Bonkhofer et al. (2019)
ATAC-seq	Whole embryo	1-somite stage	WT and cloche	N/A	Marass et al. (2019)
sc-ATAC-seq	Whole embryo	24 hpf	WT and cloche	N/A	McGarvey et al. (2022)
Histone modification studies					
ChIP-seq on H3K4me1 and H3K4me3	Whole embryo	24 hpf	WT	N/A	Aday et al. (2011)
ChIP-seq on H3K4me1, H3K27ac and H3K4me3	Whole embryo	Dome, 80% epiboly, 24 p, 48 hpf	WT	N/A	Bogdanovic et al. (2012)

The chromatin state state of endothelial cells was also studied by Dobrzycki et al. (Dobrzycki et al., 2020) (Table 2). They used bulk ATAC-seq on cells sorted by Fluorescent Activated Cell Sort (FACS) from the *Tg(kdrl:GFP)* line*,* comparing chromatin state in the GFP-positive blood and hemogenic endothelium and GFP-negative cell populations at 26 hpf. Peak validation showed the GFP-positive population was enriched for endothelial TFs such as ERG, ETS, ETV and FLI (Dobrzycki et al., 2020). Similarly, a study by Bonkhofer et al. used a combination of the transgenic lines TgBAC*(runx1P2:Citrine)*; *Tg(kdrl:mCherry),* FACS and ATAC-seq to profile the changes in chromatin organisation in the hemogenic endothelium and aortic roof endothelial cells at 29 hpf (Bonkhofer et al., 2019). Although the main focus of both these studies was to decipher the regulation of hemogenic endothelium specification, they are an outstanding resource to study the chromatin state changes in the non-haemogenic endothelial cells as well.

This comparative approach has been successfully used in other studies. Both Shin et al. (2019) and Dobrzycki et al. (2020) identified the same *gata2a* intron 4 enhancer on the basis of chromatin accessibility. The element identified drives reporter expression in the endothelium until 3 dpf, and is subsequently limited to the valve in the facial lymphatic (Shin et al., 2019; Dobrzycki et al., 2020). Such studies show the potential of this approach in identifying tissue-specific enhancers, as well as its robustness across independent experimental approaches.

The identification of gene expression heterogeneity in endothelial cells from single cell transcriptomics studies in mouse (Kalucka et al., 2020; Pasut et al., 2021) suggests the possibility that big cell populations such as AECs, VECs and LECs hide a yet uncovered heterogeneity of subpopulations, each characterized by different expression and epigenetic profiles mirroring their different environmental requirements and functions. Single cell ATAC-seq (scATAC-seq) allows us to investigate this heterogeneity, and dissect the minute complexity of gene expression regulation on a single cell level. The technique has been so-far developed in mammals (Buenrostro et al., 2015; Cusanovich et al., 2015), where it has been used to identify novel endothelial-specific enhancers (Pijuan-Sala et al., 2020) as well as enhancers active in the development of the hematopoietic lineage (Zhu et al., 2020). Recent work in zebrafish has applied this technology to hematopoietic stem and progenitor cells (HSPCs) (Avagyan et al., 2021). Works from McGarvey et al. (2022) investigated changes in chromatin modification on a single cell level between the *cloche* mutant and wild-type cells. *cloche* mutants carry a mutation in the transcription factor Npas4l (Reischauer et al., 2016) and display a complete absence of vasculature, blood cells, and endocardium (Stainier et al., 1995). In addition to providing valuable insights into molecular changes in *cloche* mutants, this study represents the first characterization of endothelial chromatin state at a single cell level. Work from the Hogan lab (Grimm et al., 2022, preprint) applied a combination of scRNA-seq and scATAC-seq in mutant strains to identify changes in chromatin stability and transcriptional outputs downstream of Prox1, the chief regulator of LECs identity. The potential of scATAC-seq for characterizing the differences in *cis*-regulation between closely related yet different cell populations, as well as the possibility of integrating such data with other single cell databases such as RNA-seq (Ranzoni et al., 2021), makes it one of the most promising techniques in the field, and opens exciting perspectives for future studies.

## Characterizing *Cis*-Regulatory Sequences Identity: Commonly Used Tools in Vascular Biology

*Cis*-regulatory activity can be identified due to characteristic histone modifications, which are a readout of the chromatin state and can be used to identify CREs or to distinguish between their different subtypes (Chen et al., 2012; Fernández and Miranda-Saavedra, 2012). Histones are proteins that form hetero-octamers around which the genomic DNA is wrapped, creating a structure called nucleosome. Histones can be subjected to post-transcriptional modifications, such as methylation or acetylation. The type of histone modifications correlates with functions and activation states of the *cis*-regulatory sequences they mark. For example, in many animals, including zebrafish, the presence of a trimethylated lysine 4 on histone 3 (H3K4me3) is associated with active promoter regions (Bernstein et al., 2002; Santos-Rosa et al., 2002; Bernstein et al., 2005; Wardle et al., 2006) while an enrichment for monomethylated lysine 4 (H3K4me1) marks the presence of an enhancer (Heintzman et al., 2007). Acetylated lysine 27 of the same histone (H3K27ac) is also enriched in enhancer sequences, with the key difference that it specifically marks active enhancers, while H3K4me1 can mark both poised and active enhancers (Creyghton et al., 2010; Bonn et al., 2012). Histone modifications can therefore be used to predict both promoter and enhancer activity.

A number of studies have uncovered specific factors involved in histone modifications that are essential for the correct development of the vasculature in zebrafish. Protein Arginine Methyl Transferase 5 (Prmt5) promotes chromatin looping, allowing accessibility to transcription factors required for vascular morphogenesis (Quillien et al., 2021). In the same way, the histone acetyltransferase P300 has been shown to be involved in blood vessel formation in zebrafish (Fish et al., 2017) and to be recruited by ERG to endothelial genes *in vitro* (Kalna et al., 2019), further indicating the complex interplay between chromatin regulators and tissue specific gene expression. Additional endothelial-enriched epigenetic regulators, including factors involved in histone modification and chromatin remodeling, have been identified using RNA-sequencing of isolated zebrafish endothelial cells (Matrone et al., 2021). This work validated one of the histone methyltransferase, Prdm16, as being necessary for zebrafish angiogenesis. Further evidence for the importance of correct histone methylation is supported by the role of the histone demethylases Kdm4a and Kdm4c in zebrafish vascular formation (Wu et al., 2015). DNA methylation is another marker associated with activation of gene expression, nucleosome organization and histone modifications (Robertson, 2002) and in zebrafish can be visualized by the recently developed transgenic model zebraRDM, which takes advantage of a fluorescent protein fusion with a methyl-CpG binding domain (Zhang et al., 2017). This line promises to be a powerful tool to study the dynamics of DNA methylation in real time, working as a complementary tool to the standard methods for identifying histone modifications.

The most common method used to identify histone modifications is chromatin immunoprecipitation sequencing (ChIP-seq) using antibodies specific for the modified histones. The histones of interest are immunoprecipitated together with the fragments of DNA bound to them after chromatin shredding, and then sequenced. Histone modification studies on whole-embryo samples are available for zebrafish. However, the lack of tissue specificity limits the utility of such databases to already known endothelial genes. One of these sets was generated from embryos at 24 hpf and maps the presence of H3K4me1 and H3K4me3 histone modifications (Aday et al., 2011). Although some genes expressed specifically in the endothelium at 24 hpf, such as *dusp5*, *fli1b* and *plxnd1*, are marked by both histone modifications in the promoter region, no vascular enhancer has been identified based on this database, possibly because of the dilution of tissue-specific signals. A second genome-wide histone modification dataset comes from a study by Bogdanovic et al. (2012) which generated histone modification tracks for H3K4me1, H3K4me3 and H3K27ac at four different time points in development, including 48 hpf (Table 2). This database has later been used to successfully identify a *notch1b* enhancer driving arterial expression in zebrafish (Chiang et al., 2017), suggesting that despite its limitations, it can be used to retrieve vascular-specific enhancers. Data from the DANIO_CODE consortium (Baranasic et al., 2021, preprint) provides whole-body and a number of tissue-specific ChIP-seq datasets. The technique was successfully used on mouse and cultured endothelial cells in multiple studies (Table 1), and recently it has been paired with transcriptomic and DNA methylation analysis to investigate the mechanisms underlying the segregation of lymphatic and blood endothelium in humans (Tacconi et al., 2020).

An alternative method to histone ChIP-seq for identifying active promoter elements is the combination of Cap Analysis of Gene Expression sequencing (CAGE-seq) with ATAC-seq, an approach that has been used in zebrafish (Nepal et al., 2013). CAGE-seq maps the position of TSS by sequencing the mRNAs containing the 5′ cap. As TSS can only be found in promoters, it allows the identification of these CREs and, by exclusion, of enhancers as well. Although currently CAGE-seq has not been used for endothelial zebrafish cells, it has been successfully applied to culture blood and lymphatic endothelial cells, leading to the discovery of the role of the transcription factor Mafb during lymphangiogenesis (Dieterich et al., 2015), a function that is conserved in zebrafish (Koltowska et al., 2015b; Dieterich et al., 2015; Rondon-Galeano et al., 2020). Combining CAGE-seq with gene expression profiling such as RNA-seq or microarrays is a useful method to identify gene expression changes downstream of epigenic modification, and studies using this approach in endothelial cells *in vitro* are summarized in Table 1.

Recently, CUT&RUN and CUT&Tag have emerged as a more robust alternatives to ChIP-seq. In Cleavage Under Targets and Release Using Nuclease (CUT&RUN) (Skene and Henikoff, 2017), permeabilized cells are immobilized with magnetic beads and the desired DNA-binding protein is targeted with antibodies, which are recognized by a ProteinA-MNase fusion construct. The MNase moiety cleaves the DNA, releasing the fragments in the solution, from which they are collected and sequenced. Compared with ChIP-seq, CUT&RUN require less input (fewer cell numbers) and reduces background noise, requiring less-deep sequencing and consequently cutting costs. Despite its recent development, CUT&RUN has already been used successfully in zebrafish, targeting both TFs (Campbell et al., 2021; Ye et al., 2021) and histone modifications (Akdogan-Ozdilek et al., 2021; Ye et al., 2021).

A further development of the CUT&RUN technology is Cleavage Under Targets and Tagmentation (CUT&Tag) (Kaya-Okur et al., 2019). In this technique, the ProteinA is fused to a Tn5 transposase, able to ligate sequence adapters directly to the cut DNA, eliminating the need for library preparation. Because of the high affinity and high activity of the core enzyme, CUT&Tag can be used with extremely low cell input, including single cells (Kaya-Okur et al., 2019). In zebrafish, CUT&Tag has been successfully used to investigate the localization of the histone variant H2A.Z in shield stage and 24 hpf embryos (Akdogan-Ozdilek et al., 2021).

Together, these recently developed techniques promise to revolutionize the field of chromatin characterization, making the profiling of DNA-binding elements easy to perform and reducing cost and time investments.

Together with ATAC-seq, techniques such as ChIP-seq, CAGE-seq, CUT&RUN and CUT&Tag can allow us to draw a picture of the chromatin state in endothelial cells and its variation both in time and within subpopulations, offering an unprecedented level of insight into the epigenetic changes underlying endothelial development.

## The power of genomic conservation in enhancer identification - Application in endothelial cells

At the beginning of the millennium, with the advent of whole-genome sequencing of multiple animal species, it became evident that many of the previously described enhancer elements overlapped with areas of high sequence conservation between species (for an exhaustive review of the early work on conserved enhancers, see Kikuta et al., 2007).

The DNA sequences that are involved in gene expression regulation include enhancer elements, containing the DNA motifs recognized by the different transcription factors. These motifs are not free to mutate as much as the surrounding non-coding DNA, leading to enhancer sequences often being more conserved than their surroundings (Plessy et al., 2005; Visel et al., 2007). Interestingly, recent reports have shown that the tissue-specificity of H3K4me1-marked enhancers can be conserved between phylogenetically distant organisms despite lacking sequence conservation (Wong et al., 2020). However, enhancers associated with genes involved in embryonic development are often highly conserved (Woolfe et al., 2004). Thus, analysis of conserved non-coding elements (CNEs) represents a quick and useful approach to the identification of enhancers, complementary to histone marks and chromatin accessibility (discussed in the *Defining the Chromatin State of Endothelial Cells and Characterizing CREs Identity: Commonly Used Tools in Vascular Biology* sections of this review).

Tools to identify CNEs are available *via* the USCS genome browser, in the form of tracks that report sequence conservation among teleosts and tetrapods, such as the Multiz Alignment and Conservation on Zn9 (Raney et al., 2014). Alternative customizable methods that allow local alignment between species include mVISTA (Mayor et al., 2000; Frazer et al., 2004) or MultiPipMaker (Schwartz et al., 2000) alignment programs. As the sequences are input by the user, any annotated genome of interest can be used for the alignment. Local synteny, which is the topological conservation of the loci surrounding the gene of interest in different organisms (Thomasova et al., 2002; Engstrom et al., 2007), is an important parameter to consider when identifying conserved enhancers. If the loci have maintained their relative position, without major transpositions of DNA material, it can be inferred that the non-coding regions in between them can be considered homologous and can therefore contain conserved regulatory sequences. It is important to highlight that with the immense progresses in computational capabilities, methodologies which use deep learning and convolutional neuronal networks to predict enhancer identity based on DNA sequence are being developed (de Almeida et al., 2021, preprint; Min et al., 2017; Yang et al., 2017). However, these methods have yet to be used in vascular biology.

The traditional sequence conservation analysis has been employed in the discovery of a number of endothelial-specific enhancer elements. Bussmann et al. investigated the presence of CREs of *flt1*, a gene expressed in the AECs, by comparing the surrounding regions in 11 vertebrate species, and identified two enhancers driving arterial expression (Bussmann et al., 2010). Additional enhancer elements that are conserved among vertebrates have been identified for a number of endothelial genes, including *etsrp* (Veldman and Lin, 2012), *gata2a* (Dobrzycki et al., 2020) and *notch1b* (Chiang et al., 2017).

The identification of endothelial enhancers in comparative studies with mouse has also led to further dissection of the signaling pathways regulating blood vessel development. Elegant *in vivo* investigations of the enhancer elements of *Notch* and the ligand *Dll4* in mouse and zebrafish position SoxF transcription factors upstream of Notch in the regulation of arterial identity (Sacilotto et al., 2013; Chiang et al., 2017). The venous identity is dependent of COUP-TFII and Ephb4, and the functional dissection of their enhancers in zebrafish and mouse revealed a requirement for ETS for tissue specific gene expression (Neal et al., 2021). Furthermore, the characterization of two *flk1* enhancers, presenting binding site for GATA and ETS, provided a direct link between the Notch and Vegf signaling pathways (Choi et al., 2007; Becker et al., 2016).

Although CNEs likely represent only a small subset of active enhancers, their study can provide important insight in the most conserved aspects of vascular development, offering hints at homologous processes taking place in humans and other Vertebrates.

## From Chromatin Architecture to Local Regulation

An important, yet less studied, aspect of spatiotemporal regulation of gene expression is 3D genome organization, a process that brings inter- or intra-chromosome regions together to activate or repress gene expression. As the regions of interaction often mark underlying enhancer activity, studying chromatin architecture provides indications on the potential presence of long-range enhancers. These are CREs located hundreds or more kb away from the promoter they regulate, such as, for example, an *Shh* enhancer located in the LMBR1 locus, more than 1 Mb away from the promoters it interacts with (Lettice et al., 2003), or the two regulatory regions associated with *Myc* function in craniofacial development and located at more than 1 Mb from the locus (Vural Uslu et al., 2014). The significant distance between this class of enhancers and the promoters they interact with, as well as the possible presence of several loci within this distance, makes it extremely difficult to identify these CREs with the methods discussed so far in this review.

Chromatin 3D conformation is commonly investigated using technologies such as Hi-C (Belton et al., 2012), which is based on the principle that enhancer-promoter interactions require the DNA to be in close contact spatially. Briefly, chromatin is cross-linked in its 3D conformation with formaldehyde, and then shredded. Fragments of DNA interacting with each other will form hybrid structures, which are then biotinylated, ligated and sequenced. By mapping the “hybrid” sequences to two separate regions of the genome, the technique allows the reconstruction of long-range genomic interactions. This approach is undoubtedly useful to reconstruct the chromatin architecture across the genome and to identify topologically associated domains, as well as long- and short-range genomic interactions. However, it is limited in its utility in predicting enhancer activity and linking the interaction to a specific gene. These limitations can be partially overcome by combining the methodology with other epigenomic tools such as ATAC-seq or CUT&Tag. In addition, related techniques such as Chromatin Interaction Analysis by Paired-End Tag sequencing (ChIA-PET) and HiChIP (Fullwood et al., 2009; Mumbach et al., 2016) allow the detection of long-range DNA interaction mediated by specific proteins. For example, interactions involving the RNApolII can reveal the presence of a long-range enhancers interacting with a promoter (Li et al., 2012). These techniques have been successfully used in zebrafish (Franke et al., 2021), as well as in human endothelial cell samples (Papantonis et al., 2012; Nakato et al., 2019; Higashijima et al., 2020; Ma et al., 2022), and have led to the identification of a distal endothelial KLF4 enhancer (Maejima et al., 2014). The importance of protein-protein interactions (PPIs) in mediating the contact of long-range genomic regions has been shown by Weintraub et al. in their work on enhancer-promotor loops. This study identified a role for the transcription factor Ying Yang 1 (YY1) in facilitating and supporting the promoter-enhancer contacts, which have a functional role in regulating gene expression (Weintraub et al., 2017). Although only a few studies have investigated long-range enhancers, the recent progresses in methods that incorporate PPIs into genome-wide detection of enhancer-promoter interactions promise a rapid developemnt in this field (Wang et al., 2021). Together, the technological advances in epigenomics provide an open platform to unveil the complexity underlying long-range DNA interactions.

## The Proof of the Pudding*—In Vivo* Activity Testing of Enhancers in Zebrafish

Zebrafish is an excellent model to test the activity of enhancers *in vivo*, due to its aptitude to transient expression of reporter constructs and genetic manipulation. Moreover, stable enhancer lines have been widely used in zebrafish as endothelial-specific reporters (Table 3). Testing of enhancer activity *in vivo* is a useful tool that can be used to further understand gene expression regulation, as exemplified by the identification of the regulatory loop between SoxF and Vegfd in blood vessel formation (Duong et al., 2014).

**TABLE 3:** CRE endothelial reporter lines in zebrafish. Summary of the endothelial specific CREs used in transgenic zebrafish lines. CRE typology and tissue specificity are reported.

**Gene**	**Element name**	**Element type**	**Element position**	**Publication**	**Endothelial expression**	**Other tissues**	**Examples of generated lines**	**Publications**
*DII4*								
	*DII4in3*	Mouse enhancer	Mouse intron 3	Sacilotto et al. (2013)	Arterial endothelium	Not reported	*Tg(Dll4in3:GFP)*	Sacilotto et al. (2013)
	*DII4-F2-E1b*	Mouse enhancer	Mouse intron 3	Wythe et al. (2013)	Arterial endothelium	Not reported	*Tg(Dll4-F2-E1b:GFP)*	Wythe et al. (2013)
*Ephb4*								
	*Ephb4-2*	Mouse enhancer	Around −2 kb from intron 1	Neal et al. (2019)	Venous endothelium	Not reported	*Tg(Ephb4-2:GFP)*	Neal et al. (2019)
*Etsrp*								
	*−2.3etsrp*	Enhancer	−2.3 kb upstream	Veldman and Lin (2012)	Endothelium before 36hpf, then aortic arches	Not reported	*Tg(−2.3etsrp:gfp)* ^*zf372*^	Veldman and Lin (2012)
*fli1a*								
	*fli1a*	Promoter	−15 kb to exon 1	Lawson and Weinstein (2002)	Endothelium	Neural crest-derived tissues	*Tg(fli1a:EGFP)* ^*y1*^	Lawson and Weinstein (2002)
							*Tg(fli1a:pecam1-EGFP)* ^*ncv27*^	Ando et al. (2016)
							*Tg(fli1a:B4GALT1-mCherry)*	Kwon et al. (2016)
	*fli1a*	Promoter	−1 to +6 kb	Lawson et al. (2001)	Endothelium	Neural crest-derived tissues	*Tg(fli1a:nEGFP)* ^*y7*^	Roman et al. (2002)
	*fli1ep*	Enhancer/promoter fusion	enhancer (+2.2 to +3.2 kb) + promoter (−0.9 kb to exon 1)	Villefranc et al. (2007)	Endothelium	Neural crest-derived tissues	*Tg(fli1ep:dsredex)* ^*um13*^	Covassin et al. (2009)
							*Tg(Fli1ep:Lifeact-EGFP*	Phng et al. (2013)
							*Tg(fli1a:H2B-mCherry)*	Yokota et al. (2015)
*flt1*								
	*Flt1_9a*	Enhancer	Not specified	Bussmann et al. (2010)	Arterial endothelium, weakly veins	Not reporter	*Tg(flt1_9a_cFos:GFP)*	Kaufman et al. (2015)
	*−0.8flt1*	Enhancer/enhancer/promoter fusion	Not specified	Bussmann et al. (2010)	Arterial endothelium	Not reporter	*Tg(−0.8flt1:RFP)* ^*hu5333*^	Bussmann et al. (2010)
*flt4*								
	*−6.6flt4*	Promoter	−6.6 kb to exon 1	Hogan et al. (2009b)	Blood endothelium before 48hpf	Not reporter	*Tg(-6.6flt4:YFP)* ^*hu4881*^	Hogan et al. (2009b)
*gata2a*								
	*gata2a-i4*	Enhancer	Intron 4	Dobrzycki et al. (2020)	Endothelium	Endocardium	*Tg(gata2a-i4-1.1 kb:GFP)*	Dobrzycki et al. (2020)
	*gata2aECE*	Enhancer x6	Intron 4	Shin et al. (2019)	Endothelium at 2dpf, lympatic valve at 14dpf	Not reported	*Tg(gata2aECE:nsfGFP)* ^*um2 91*^	Shin et al. (2019)
*kdrl/flk1*								
	*kdrl/flk1*	Promoter	−6.5 kb to exon 1	Jin et al. (2005)	Blood endothelium	Not reporter	*Tg(kdrl:EGFP)* ^*s843*^	Jin et al (2005)
							*Tg(kdrl:NLS-EGFP)*	Blum et al, (2008)
							*Tg(kdrl:Hsa.HRASmCherry)* ^*s896*^	Chi et al. (2008)
	*kdr-l*	Promoter	−6.8 kb to exon 1	Hogan et al. (2009a)	Blood endothelium	Not reporter	*Tg(kdr-l:ras-cherry)* ^*s916*^	Hogan et al. (2009a)
	*Flk1in10*	Mouse enhancer	Mouse intron 10	Becker et al. (2016)	Blood endothelium before 48 hpf, then restricted to arteries	Not reported	*TgFlk1in10:GFP)*	Becker et al. (2016)
*lyve1b*								
	*−5.2lyve1b*	Promoter	−5.2 kb to exon 1	Okuda et al. (2012)	Venous and lymphatic endothelium	Not reporter	*Tg(−5.2lyve1b:DsRed)* ^*nz101*^	Okuda et al. (2012)
							*Tg(−5.2lyve1b:Venus)* ^*uq16bh*^	Bower et al. (2017)
							*Tg(lyve1b:Kaede)* ^*nz102*^	Eng et al. (2019)
*Mafbb*								
	*mafbbEnh*	Enhancer	7.8 kb downstream	Quillien et al. (2017)	Venous endothelium	Not reported	*Tg(mafbbEnh-basP:egfp)*	Quillien et al. (2017)
*Mef2c*								
	*mef2c-F10*	Mouse enhancer	Mouse intron 4–5	De Val et al. (2008)	Endothelial cells	Not reported	*Tg(mef2c-F10-GFP)*	De Val et al. (2008)
*mrc1a*								
	*mrc1a*	Enhancer/promoter fusion	Enhancer (intron 19) +promoter (−1.9 kb to exon 1)	Jung et al. (2017)	Venous and lymphatic endothelium, before 3 dpf Some expression in arterial endothelium	Myeloid cells	*Tg(mrc1a:egfp)* ^*y251*^	Jung et al. (2017)
*notch1b*								
	*notch1b-15*	Enhancer	−15 kb upstream	Chiang et al. (2017)	Arterial endothelium	Not reported	*Tg(notch1b-15:GFP)*	Chiang et al. (2017)
*nrp1b*								
	*nrp1bEnh*	Enhancer	34.5 kb upstream	Quillien et al. (2017)	Blood endothelium	Not reported	*Tg(nrp1bEnh-basP:egfp)*	Quillien et al. (2017)
*Tie2*								
	*Tie2*	Mouse Enhancer/Promoter fusion	Enhancer (10 kb intron 1) + promoter (−2.1 kb to exon 1)	Schlaeger et al, (1997)	Early endothelium	Hematopoietic mesoderm, endocardium	*Tg(Tie2:EGFP)* ^*s849*^	Motoike et al. (2000)
*tmem88a*								
	*tmem88aEnh*	Enhancer	3.8 kb upstream	Quillien et al. (2017)	Blood endothelium	Not reported	*Tg(tmem88aEnhbasP:egfp)*	Quillien et al. (2017)

The most common way to test enhancer activity is to clone the putative sequence upstream of a minimal promoter followed by a fluorescent reporter in a plasmid backbone containing tol2 sites for transgenesis. The construct is then injected into 1-cell stage zebrafish embryos. Generation of the stable lines is often advisable to confirm the expression pattern.

When testing enhancer activity, it is important to remember that regulatory elements often act in concert. Therefore, an element unable to drive tissue-specific expression could still be involved in regulation, but not be sufficient to drive the reporter on its own.

A way to circumvent the issue of recapitulating the real regulatory landscape is to test the endogenous enhancer activity *in situ.* However, this approach remains challenging as the tools that efficiently generate such reporters are limited. Successful endogenous tissue specific enhancers lines have been generated by enhancer trapping (Balciunas et al., 2004; Kawakami et al., 2010), including some for endothelial genes such as *tal1* (Veldman et al., 2013). This method takes advantage of random insertions of GFP expression constructs into the genome, followed by screening of the expression pattern. Therefore, it offers more of a “forward genetic” approach to enhancer screening (Kikuta et al., 2007; Kawakami et al., 2017). The rapid expansion of CRISPR technologies for knock-ins (Kimura et al., 2015; Albadri et al., 2017) is a promising alternative to the previous approaches, and a number of gene and promoter mutant lines have been generated with this technique (Ota et al., 2016; Kesavan et al., 2017; Eschstruth et al., 2020). However, this method has not yet been tested for endothelial enhancers. The progresses in knock-in technology are also opening the possibility of working with conditional mutants in zebrafish. The recent advances in the establishment of the CRE/lox system in this model (Burg et al., 2018; Kesavan et al., 2018) are leading the way for the development of conditional mutant lines. In this context, enhancers driving CRE in a subset of the overall gene expression will provide an exceptional tool to generate tissue-specific conditional mutants.

Enhancer expression is often weak and spatially limited. Therefore, screening for positive embryos can be demanding. To accelerate the process, a preselection of positively injected embryos can be performed by the introduction in the plasmid backbone of a second reporter construct, such as e.g., αcry:GFP (Quillien et al., 2017), driving GFP in the lens. Such constructs are often chosen because they drive easily-identified expression in a tissue that is not of interest for the study. The Gomez-Skarmeta group has developed the ZED vector (Bessa et al., 2009), a tool specifically designed to test enhancer activity, containing the XCA:DsRed muscular selection marker and a Gateway cloning site for the enhancer in front of a basal promoter:GFP element.

CRISPR/Cas9 genome editing has also been successfully used to test the functional relevance of an enhancer and whether it is necessary to drive endothelial gene expression. In recent years, we have seen multiple examples of functional testing of vascular enhancers *in vivo.* Deletion of a *notch1b* enhancer driving reporter expression in the dorsal aorta and intersegmental arteries led to a reduction in the expression of the reporter in these two tissues, but not in other normally expressing *notch1b* (Chiang et al., 2017). Similarly, deletion of the i4 enhancer of *gata2a* caused a reduction of the expression of this gene in the endothelium, but not in other tissues (Shin et al., 2019; Dobrzycki et al., 2020). It has to be noted however that in all these cases the effect on vascular morphology were small (Dobrzycki et al., 2020), limited to very specific structures (Shin et al., 2019), or connected to maternal effects (Chiang et al., 2017). As enhancer activity often has very narrow spatiotemporal restrictions, the effects of enhancer loss can be minimal, which can make the identification of the mutant phenotype difficult. However, the increased specificity of the phenotype can provide unique insight into more specific regulatory mechanisms in developing tissue, which could not be observed in more traditional gene knockouts. Thus, enhancer deletion remains an important tool to determine the regulatory networks driving tissue and organ formation.

## Conclusion

The recent technological advances in epigenomics opened new and exciting avenues to study the complexity of gene regulation on multiple levels, from local to genome-wide, promising to uncover new paradigms in gene regulation and to reveal an unsuspected complexity in the development of tissues, including vascular networks. This comprehensive understanding linking the cellular morphological transformations with the complex mechanisms regulating gene expression is a long-awaited progress in biology.

The recent developments in single-cell genomics, such as scATAC-seq, scCUT&RUN/Tag and scChIP-seq, hold the potential for uncovering undescribed mechanisms of regulation underlying the cellular and molecular heterogeneity of vascular networks. At the same time, emerging technologies in the study of 3D chromatin architecture and long-distance regulatory interactions, such as Hi-C, have now been applied to zebrafish for the first time (Yang et al., 2020), and are opening the possibility to study enhancer regulation of gene expression on a global scale.
